# Injuries among Artisanal and Small-Scale Gold Miners in Ghana

**DOI:** 10.3390/ijerph120910886

**Published:** 2015-09-02

**Authors:** E. Kyeremateng-Amoah, Edith E. Clarke

**Affiliations:** Occupational and Environmental Health Unit, Ghana Health Service, PMB, Ministries, Accra, Ghana; E-Mail: clarke.edith@gmail.com

**Keywords:** artisanal small-scale gold mining, injuries, accidents

## Abstract

Artisanal and small-scale gold miners are confronted with numerous hazards often resulting in varying degrees of injuries and fatalities. In Ghana, like many developing countries, there is paucity of information on the causes and nature of the accidents that result in the injuries. The study was a retrospective, cross sectional type that examined the records of injuries of artisanal and small-scale gold miners presented to the emergency department of a district hospital in the Eastern Region of Ghana from 2006 to 2013. The causes, types, and outcomes of reported injuries were analyzed for 72 cases. Occurrences of mining accidents reported in selected Ghanaian media during the year 2007–2012 were also analyzed to corroborate the causes of the accidents. Fractures and contusions constituted the most frequently occurring injuries, with collapse of the mine pits and falls being the most frequent cause of accidents reported both by the hospital and media records. This study shows that though varied degrees of injuries occur among the miners, the potential for serious injuries is substantial. Measures to reduce the incidence of injuries and fatalities should include education and training on the use of safe working tools and means of creating a safe working environment.

## 1. Introduction

It is estimated that about 13 million people work in artisanal and small-scale mining (ASM) and an estimated 100 million people depend on it for their livelihood worldwide [1]. This sub-sector of mining is estimated to contribute about 13% of the world’s gold production and about 15% of the world’s non-fuel minerals [2]. It remains a major source of income and has been described as a substantial means of generating income in many developing countries [3].

Ghana is the eighth leading producer of gold in the world and the second largest producer in Africa [4,5]. In 2012, 27% of the government’s tax revenues were attributable to the mining industry, with gold receipt revenues amounting to approximately more than $5.6billion from 4.3million ounces [6].ASM contributes about one third (34%) of the national gold production, thus serving as a major component of the economy [7]. The Precious Minerals Marketing Company (PMMC), which is the government’s agency for trading and exports of precious minerals in the country, increased its total purchases and exports of gold from small-scale miners from235, 787 ounces in 2011 to 316,699 ounces in 2012 [8]. The 2012 performance of gold in the country was about 34% higher than the amount recorded in the previous year. Over the same period, Asap Vasa, a private precious mineral trading company, significantly increased its purchases and exports of gold from small-scale miners from 10,173 ounces to 40,794 ounces, a 301% increase [8]. The great demand for gold both locally and internationally coupled with low-income levels, increasing unemployment rate, and poverty makes artisanal and small-scale gold mining (ASGM) an attractive venture for many to engage in. However, the individuals who participate in ASGM often lack the training, skills, and knowledge needed to practice safely [9]. The small-scale gold mining sector provides employment for a large proportion of otherwise unemployed individuals in Ghana. Thus, small-scale gold mining is of tremendous economic importance in Ghana, and many other developing countries, especially in places where alternative livelihoods are limited, though a number of public health hazards exist in the sector that pose challenges [10,11].

ASM encompasses the exploitation of the mineral ore deposits, which is characterized by the usage of relatively simple tools at low production levels, is often undercapitalized and occurs within informal settings [12]. In Ghana, the number of ASM is estimated to be between 180,000 to 200,000 [13].While some operatives are now registered as small-scale miners, the majority still operates illegally and is known as “*galamsey*’’ (meaning “gather and sell”) [9]. The percentage of mines that operated illegally is shown to be between 40%–50% in 1999 [1]. In terms of operational methods, it is difficult to distinguish between the two groups of miners as they use similar methods. Though ASM in Ghana is generally characterized by intense manpower with rudimentary equipment such as shovels, pickaxes, and poor methods of extraction, some operators have engaged the use of excavators, bulldozers, and other sophisticated equipment [12,14]. It is believed that the rapid introduction of mechanization without the appropriate safety training for such machinery has contributed significantly to the risks from this type of mining. Some of the high risk operations cited includes blasting without training and pneumatic drilling without dust control [12]. The mine owners or organization usually has the responsibility of providing for the safety of the miners.

In Ghana’s ASM industry, major health risks which have been identified include exposure to dust which may cause silicosis from inhalation of free crystalline silica [11]. Additional risks are exposure to mercury, carbon monoxide; noxious gases such as sulphur dioxide and nitrous fumes from explosive dynamite blasts. Other hazards include vibration from machinery, consequences of poor ventilation (heat, humidity, lack of oxygen), effects of over-exertion, inadequate workspace and inappropriate equipment [1].The effects of the exposure to these hazards, especially among minors (aged under 18 years), include constant headaches, joint disorders, visual problems and dermatological, muscular, and orthopedic ailments [15].

The ILO estimates that the workplace fatality rate of small-scale miners is about 90 times higher than that for large-scale mines in industrialized countries [16]. Informal mining poses even more hazards than what may be found in a highly organized and/or regulated large-scale operation. For example, the International Labour Organization estimates that non-fatal accidents are 6–7 times more common in informal mining operations when compared to large-scale operations [16].

Mercury use has been linked to the artisanal mining of gold in West Africa [17]. The main environmental problems associated with artisanal gold mining activities in the Ghana are mercury pollution from gold processing, ecosystem destruction, and environmental degradation [18].

### Justification for the Study

There is widespread activity of ASGM activities in Ghana, and the eastern region of the country is home to several of such activities. The Holy Family Hospital in Nkawkaw which is the only district hospital in the Kwahu West Municipality lies within the catchment area of surrounding towns and villages where these activities are prevalent. It therefore serves as one of the major centers where patients seek medical attention and where cases requiring more than primary care are referred for attention. Despite the widespread ASGM activity in Ghana, little is known about the types and prevalence of accidents and injuries. Given the location of the health facility, it is likely that some of the injuries among the ASGM miners within the area would report to the facility for medical care. The objective of the study therefore was to explore the nature of injuries reported by artisanal and small-scale gold miners by examining their hospital records at the district hospital in the eastern region of Ghana.

## 2. Methodology

This was a retrospective, cross sectional study that examined the hospital records of injuries presented to the emergency department of a district hospital, in the Eastern Region of Ghana. The region has a rich gold ore deposit and is home to both large mining companies as well as ASGM activities. The Holy Family Hospital, which serves as the only district hospital in the Kwahu West Municipality, lies within the catchment area of surrounding towns and villages where these activities are prevalent and should have been recording increasing cases of injuries related to ASGM activities. The health system in Ghana consists of primary, secondary and tertiary care centers. The district hospital falls under the secondary care level, and thus also serves as the major referral facility for the municipality and beyond.

The period for the study was 2006 to 2013.The identified cases recorded in the emergency department’s register before the year 2006 did not have the complete information for inclusion into the study. The inclusion criteria were all cases reporting to the emergency department of the facility that involved small scale/artisanal gold miners who were diagnosed by the attending physician as having had an injury in the course of their work as miners, regardless of the outcome *i.e.*, whether these were treated and discharged, admitted, or referred to a higher facility. Inclusion cases should have the age, sex, address, occupation, diagnosis, and outcome recorded and available. The registers from the year 2006 onwards had information that fulfilled the inclusion criteria. The cases were limited to only artisanal and small scale gold miners. A total of 72 cases were identified as having met the inclusion criteria and thus were evaluated. SPSS was used for data entry and analyses. Descriptive data analysis such as the frequency, distribution and percentages of the reported incidents of mining related accidents, injuries, and fatalities were performed. A comparison of the types and outcomes of similar work related injuries among the ASGM were made by reviewing reports of mining accidents from five print media (newspaper), the national news agency, and one radio media source from 2007 to 2012. The sources were; The Daily Graphic, Ghanaian Times, Ghana News Agency, The Chronicle, Daily Guide, Accra Mail, and My Joy On-line (radio media). Google search engine was used to find the information. It yielded data from the online versions of the newspapers (The Daily Graphic and the Accra Mail), the Ghana News Agency and the radio media. The key search words were; “galamsey”,“small scale” or “artisanal gold miners”, “injuries”, “accidents” and “Ghana”. The newspapers in hard copy versions (Daily Guide, Ghanaian Times and The Chronicle)were sorted out manually according to the inclusion criteria. The inclusion criteria were any reported work related incidents involving artisanal or small scale gold miners from 2006 to 2013.

## 3. Results

### 3.1. Miner Demographics

**Figure 1:**
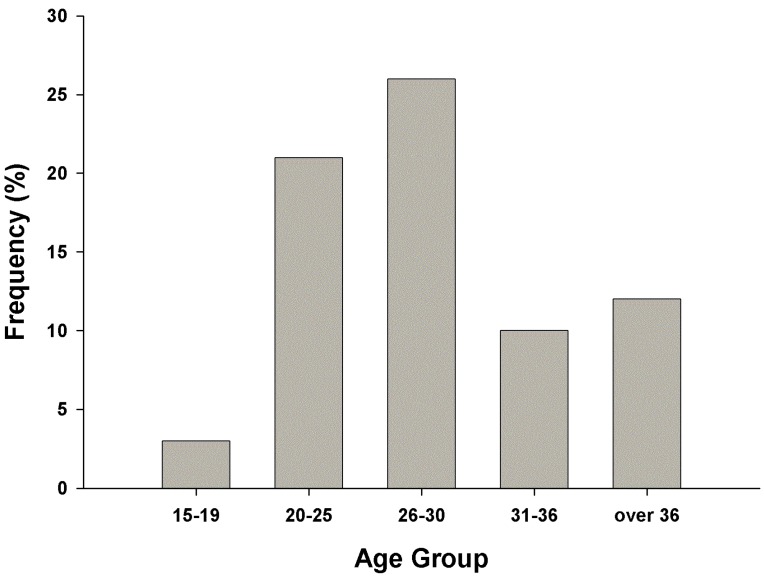
Age distribution of the injured miners.

A total of 72 cases involving injuries among miners were recorded at the hospital over the period 2006 to 2013. Their ages ranged between 15years upwards with the maximum recorded age of 45 years. The majority of the miners were aged between 20 to 30 years (Figure 1). Males accounted for over 97% of casualties studied.

### 3.2. Type of Accidents Causing Injuries

Categorizations on the causes of accidents were obtained from the physician notes based on history taken from the patients during the clinical encounter, and were stated as part of the diagnosis, which was captured in the registers. There were no records on the cause of injuries for majority of the cases (n = 51; 70.8%).For those cases on which these records existed, collapse of the mine pit was the most frequent cause (n = 9; 12.5%) of accidents resulting in injuries followed by explosive blast injuries (n = 7; 9.8%). In three of the cases studied, which involved falls at the site, two miners fell into pits,and one fell at site after being injured by a falling heavy metallic object.

### 3.3. Types and Outcome of Injuries Sustained by Miners

**Figure 2:**
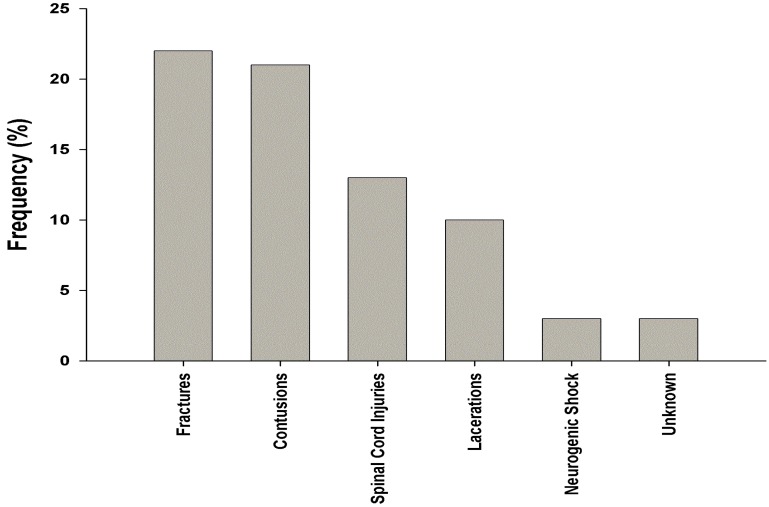
Frequency of injuries recorded for the injured miners.

Various injuries were sustained among the miners over the study period. These were contusions, lacerations, fractures, spinal cord injuries, and neurogenic shock (Figure 2).Fractures and contusions constituted the most frequently occurring types of injury accounting for about a third of reported injuries respectively. The rest were spinal cord injuries, lacerations, and neurogenic shock. Over half (n = 41) of the injured were treated in the emergency room and discharged. About a quarter (n = 20, 27.8%) of the patients were referred to seek further care and management at tertiary level facilities and10 percent (n = 7, 10%) of the injured were admitted at the hospital. Almost three percent (n = 2, 2.8%) of the injuries resulted in death. This applied to two miners who were pronounced dead on arrival at the healthcare facility.

### 3.4. Media Reports on Mining Accidents

**Figure 3:**
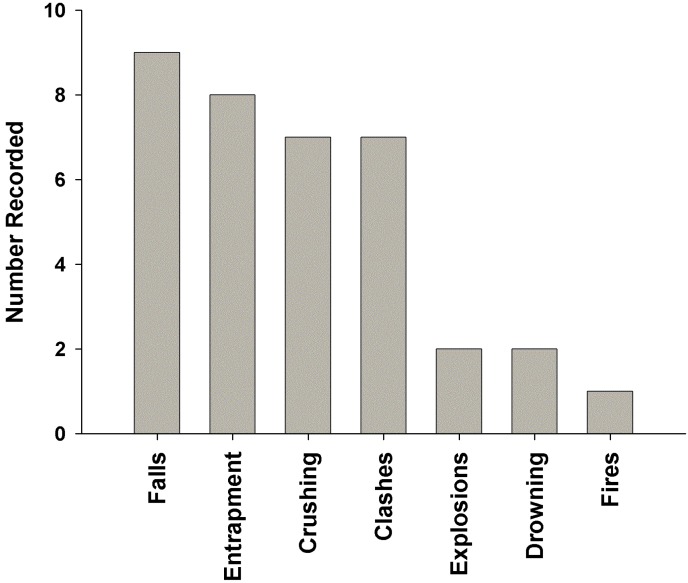
Causes of accidents reported from a review of media articles.

Media reports (from Daily Graphic, Ghanaian Times, Ghana News Agency, Chronicle, Daily Guide, Accra Mail, and My Joy On-line) from the period 2007 to 2012 provided some information on type of mining in which accidents occurred, and the underlying causes of accidents. Nearly all (97.4 %) of the reports involved persons operating in ASM, while2.6% of the accidents occurred in large-scale mining operations. Causes of accidents reported consisted of falls (n = 9, 25%), followed by entrapment from collapse of mine pits (n = 8, 22.2 %), crushing (n = 7, 19.4 %), clashes between miners and other factions (n = 7, 19.4%), drowning (n = 2, 5.6 %), explosions (n = 2, 5.6 %), and fires (n = 1, 2.8%), (Figure 3).

## 4. Discussion

In the current study, a review of hospital records revealed that the commonest types of injury reported among ASGM miners taken to the hospital were fractures (30.5%), followed by contusions (29.1%), and lacerations (14.0%). Such findings are important in shaping preventative actions and policies given that there is a paucity of studies in Ghana describing the nature of injuries and the causes of predisposing accidents, among ASGM operatives. One of the available studies by Sutherland relates to workers in large-scale formal gold mining. The study found that lacerations were the commonest type of injury, (constituting 39.3% of recorded injuries), followed by contusions (24.8%), fractures (10.2%), traumatic amputations (4.7%), burns (3.4%), and multiple injuries (2.4%) [19]. Calys-Tagoe *et al.* conducted a survey-based cross sectional study of 404 small-scale miners in the Tarkwa mining district of Ghana. They recorded a total of 121 injury episodes involving 95 miners. Upper limbs were the body part most frequently injured, with lower limbs being the second most common. Lacerations (57%) were the most common injury type [20]. Another recent study by Long *et al.* in an ASGM community in Ghana’s Upper East Region that surveyed 173 people in 2011 and 22 people in 2013 likewise found that lacerations were the most common injury type. They also reported that leg and knee injuries were the most common mining injuries [21]. Both of these newer studies found that being hit or struck by an object was the most common cause of injury [20,21]. Our study revealed that lacerations were the third most common type of reported injury following after fractures and contusions. The difference in the findings from these previous studies and ours may be attributable to the healthcare seeking behavior of the ASGM and the types of injuries that they may be willing to seek medical attention for.

It is not uncommon in the Ghanaian setting for minor cuts or ailments to be treated either at home or by other means without resorting to health facilities. The current study presents records of injuries reported by miners as indicated in the records from the health facility. These are injuries severe enough to require medical attention. Buxton indicated that attendance at hospitals and clinics among ASGM workers generally follows only after sustaining serious injury or illness [13]. In the large mining settings, it is expected that all injuries including near misses are recorded, yet even in settings with well-established reporting systems, underreporting is not uncommon; hence it is not surprising that lacerations accounted for the major type of injuries in that setting. The lacerations, whether minor or major, are expected to be recorded as opposed to the ASGM operatives who may seek self-medication for minor injuries.

Since most of the miners are migrants and move along vast tracts of land, the use of mobile healthcare services, where healthcare services are rendered close to their workplace, could facilitate the capture of injuries when they occur. In the absence of the mobile healthcare facilities, the presence of healthcare facilities within the mining communities could encourage health seeking in the event of an injury and limit underreporting. A useful surveillance system for injuries among ASGM miners at the healthcare facilities could include registers that collect routine data on their demography, job roles at the mines, causes and types of accidents as well as types of injuries. Injuries that do not report to the health care facilities could still be captured by the local/district mines’ inspectorates where available and such data could be shared with the health directorate/sector either at the district or regional level. This system of data collection however would be enhanced if the miners operate formally and are educated and encouraged to report injuries willingly

The leading underlying causes of accidents resulting in injuries among ASGM operatives in this study from the hospital records were collapse of mine pits (13%), dynamite/explosive blasts (10%),and falls (at site and into the mine pits-5%).The causes of accidents resulting in injuries as analyzed from the media sources also indicated that falls were the most common cause of accidents(25%), followed by entrapment from the collapse of mine pits (about 22%).Thus falls and collapse of mine pits are major causes of injuries among these miners (Figure 3).

### Findings from Other African Countries

Beyond Ghana, there are a handful of studies from across Central and East Africa that have found similar types of accidents and injuries in ASGM sites. In the province of Katanga, in the Democratic Republic of Congo, a study of 180 artisanal miners found that tools handling and handling of heavy loads accounted for 51.5% and 32.9%of the accidents respectively. Contusion was found to be the most common type of injury (50.5%) followed by wounds (44.4%). Physical complications/sequelae were reported by 29% of those who were injured [22]. The most frequently occurring causes of accidents which related to handling of tools and handling heavy loads, is in contradiction to the current study in which collapse of the mines is the most prevalent cause. However, contusions (which in our study were second only to fractures) are the most prevalent type of injury. Wounds, which may be a result of lacerations or crushing injuries, are also prevalent. There are therefore similarities in the injury types between Ghana and the Democratic Republic of Congo.

The underlying causes of accidents resulting in injuries have been variously described. Jennings cites the five most frequent causes as falling of rocks, lack of ventilation, misuse of explosives, lack of technical training and training on regulatory compliance and obsolete and poorly maintained equipment. There have been several reports of fatalities and severe injuries among artisanal and small-scale gold miners [1]. A study on a small number of small-scale miners in South Africa revealed numerous occupational health and safety challenges including deficiencies in risk assessments, limited access to technical expertise and hazard monitoring, risks related to dust and rock falls, deficient management systems, and the inability to comply with current health and safety standards [23].

In the Busia district of Tanzania, it was noted that fatalities and serious injuries occurring due to collapse of pit walls or underground tunnels presented a major and an immediate challenge for the ASGM operatives. It is estimated that between one and five deaths occur annually, with many others being unreported [24].Fatalities among AGSM miners in Zimbabwe have also been attributed to their re-entry into closed mines to win gold from pillars and from alluvial miners burrowing into uncompact driver beds [2]. The country has been described as one with a “disproportionately high number of fatalities” among the ASGM workers. These findings are similar to the outcome of our study in which collapse of mine pits are the most prevalent cause of injuries presented to health facilities.

In our study, 2.8% of the injuries resulted in fatalities (Section 3.3). The measures to curb such injuries and fatalities associated with ASGM may be complex and multifaceted ranging from government’s commitment, specialized interventions such as engineering solutions training and education of miners,enhancing formalization of the ASGM activities, and enhancing access to available support among others.

Though this study adds to a growing and somewhat consistent database concerning accidents and injuries in ASGM sites, there are some limitations that warrant mention. The hospital data obtained did not contain all the characteristics of the accidents and injuries of the miners. This is because the hospital records are not designed to routinely capture the causes of accidents. Therefore many of the underlying causes of the specific reported accidents and injuries were not obtained. The method of data gathering used for this study may have inherently limited the total number of injuries and deaths associated with artisanal and small scale gold mining.

The study utilized reports by the print media mainly to assess the types and underlying causes of accidents suffered by the miners during their operations. Most of the reports were made by eye witnesses to the incidents. While some of the interviewees were affected miners, others were non-miners who were present at the site when the incident occurred. Another limitation to the study was that the total number of miners exposed was not obtained. This would have made it possible to estimate what proportion of miners were involved in the accidents and affected by injuries.

Surveillance systems could help to address this by capturing the number of miners who were exposed during the period of the incident from miners who come in to seek medical care; however this approach would be subject to recall biases. The period for collection of data varies between the health records and media reports. While hospital data covered a seven-year period from 2006 to 2013, the data from the media only covered a five-year period from 2007 to 2012 year within that period. This limits the extent of comparability of data from the two sources.

Future area of research could include a study to determine whether training has any relationship on the number or rate of work related injuries occurring among the miners who are duly registered, and to compare the outcome with those who are operating without due registration.

## 5. Conclusions

The study reveals that injuries sustained by ASGM could range from minor types such as contusions to major types such fractures or spinal cord injuries, and even sometimes result in fatalities. These injuries come as a result of unsafe working conditions, notably caving in of mine pits, clashes, and explosive blasts. The exact distribution of the injury types is, however, difficult to determine fully. This is due to the fact that many miners may not report to the hospital when injured and there is a lack of standard reporting structures on injuries that occur on the job at the mines. It would be important for local health authorities to capture the causes of injuries and accidents among this group of workers when they present to the healthcare facilities.

It is evident from the study that the local health authorities do not adequately capture the reported cases of injuries. This is likely to be as a result (at least in part) of a lack of understanding of the conditions under which ASGMs operate and their injury risks. There is therefore the need for greater awareness and training of health professionals (particularly those practicing in mining areas) concerning the exposures of ASGM miners and their community members, so that they do not miss relevant symptoms presenting at the health care facilities. This will help to improve their surveillance systems and design clinical registers that take cognizance of injury types and their causes.
